# ADAMIXTURE: adaptive first-order optimization for biobank-scale genetic clustering

**DOI:** 10.1093/bioinformatics/btag236

**Published:** 2026-07-07

**Authors:** Joan Saurina-i-Ricos, Daniel Mas Montserrat, Alexander G Ioannidis

**Affiliations:** Department of Biomedical Data Science, Stanford University, Palo Alto, CA 94305, United States; Genomics Institute, University of California, Santa Cruz, Santa Cruz, CA 95060, United States; Department of Biomedical Data Science, Stanford University, Palo Alto, CA 94305, United States; Department of Biomedical Data Science, Stanford University, Palo Alto, CA 94305, United States; Genomics Institute, University of California, Santa Cruz, Santa Cruz, CA 95060, United States

## Abstract

**Motivation:**

Estimating genetic clusters from sequencing data is a fundamental task in population and medical genetics, enabling demographic inference and adjustment for population structure in association studies. ADMIXTURE, a widely used model-based clustering method, employs an accelerated Expectation–Maximization (EM) algorithm to infer population parameters; however, its computational demands scale poorly, limiting its usefulness for modern biobank-sized datasets. While recent EM acceleration strategies employing second-order quasi-Newton schemes preserve accuracy, they remain computationally intensive. Conversely, EM-free approaches that prioritize speed often compromise solution quality.

**Results:**

We introduce ADAMIXTURE, a novel optimization framework that integrates the EM algorithm with Adaptive Moment Estimation (Adam). Unlike traditional acceleration methods, ADAMIXTURE utilizes first-order gradients with adaptive learning rates derived from raw and squared moments to approximate curvature information, bypassing the computational overhead of Hessian approximations. This approach surpasses the convergence efficiency of second-order methods while maintaining the low computational complexity of first-order updates. Across simulated and large-scale empirical datasets, ADAMIXTURE demonstrates substantial reductions in wall-clock runtime and enhanced scalability compared to state-of-the-art methods, while maintaining comparable or improved inference accuracy. Its GPU implementation runs in under 2 h on half a million samples and variants, a two order of magnitude speedup over current state-of-the-art.

**Availability and implementation:**

Source code is available at: https://github.com/AI-sandbox/ADAMIXTURE.

## 1 Introduction

The rapid expansion of population-scale biobanks and sequencing initiatives has revolutionized the genomic prediction of human traits and disease risk. However, the predictive accuracy of these models is often linked to genetic ancestry, rendering the precise characterization of population structure a prerequisite for robust analysis (Martin *et al.* 2019). For decades, unsupervised ancestry inference has served as a cornerstone for correcting stratification in genome-wide association studies and reconstructing demographic histories. Yet, as datasets scale to millions of individuals and genomic variants (Bycroft *et al.* 2018), foundational algorithms increasingly face computationally prohibitive bottlenecks. Overcoming these scalability constraints is paramount for analyzing diverse biobanks and ensuring that the promise of precision medicine is equitably realized across all populations.

The standard computational framework for this task, established by STRUCTURE (Pritchard *et al.* 2000), models individuals as mosaics of fractional ancestries derived from *K* ancestral populations. These populations are inferred via unsupervised learning in a high-dimensional space defined by variant frequencies, allowing for the characterization of continuous genetic variation without reliance on subjective labels. The model operates on single nucleotide polymorphisms (SNPs), exploiting the fact that allele frequency distributions diverge among populations due to distinct demographic processes, including migration, genetic drift, and founder effects.

While the Bayesian framework introduced by STRUCTURE was seminal, its reliance on Markov chain Monte Carlo sampling imposed severe computational costs on large datasets. To mitigate this, ADMIXTURE (Alexander *et al.* 2009) reformulated the problem within a maximum-likelihood framework, employing block relaxation and Expectation-Maximization (EM) algorithms to accelerate inference. This shift significantly extended the applicability of genetic ancestry estimation and clustering. Nevertheless, with the rapid growth of large-scale genomic cohorts, the computational demands of ADMIXTURE have likewise become prohibitive, rendering it intractable for biobank-scale analysis.

In response, recent years have seen the emergence of diverse acceleration strategies, broadly categorized into likelihood-free and likelihood-based approaches. Likelihood-free methods include matrix factorization such as sNMF (Frichot *et al.* 2014), which has been superseded in biobank-scale applications by the more scalable alternating least squares (ALS) scheme in SCOPE (Chiu *et al.* 2022). Conversely, likelihood-based accelerators focus on optimizing the EM procedure itself; for instance, fastmixture (Santander *et al.* 2024) combines mini-batch EM optimization with ALS-driven initialization. Concurrently, alternative paradigms have been adapted to this domain, such as Archetypal Analysis (Gimbernat-Mayol *et al.* 2022) and deep learning approaches like Neural Admixture (Dominguez Mantes *et al.* 2023), which respectively recasts genetic ancestry inference and clustering as an autoencoder task. While these innovations have improved scalability, they often introduce significant tradeoffs between computational speed and statistical rigor.

Addressing this dichotomy, we introduce ADAMIXTURE, a novel optimization framework that accelerates the EM algorithm by integrating it with adaptive moment estimation (Adam) (Kingma and Ba 2014). Unlike prior acceleration attempts, our method leverages first-order *pseudo-gradients* to drive robust parameter updates within a single EM step. ADAMIXTURE is available in both central processing unit (CPU) and graphics processing unit (GPU) implementations. While the CPU version ensures broad accessibility, the GPU variant leverages highly parallel computation to boost scalability and throughput. The result is a powerful tool achieving state-of-the-art performance on biobank-scale datasets.

## 2 Materials and methods

### 2.1 Model formulation

This work builds upon the probabilistic framework introduced in STRUCTURE, which describes the relationship between an observed genotype matrix, *G*, and two latent parameter matrices: the individual admixture proportions, *Q*, and the ancestral allele frequencies, *F*.

The model takes as input the M×N genotype matrix *G*, where each entry $G_{ji}\in \{0,1,2\}$ contains the count of alternate alleles for SNP *j* in individual *i*, across *M* SNPs and *N* individuals. The parameter matrices are:

- **Admixture proportions (*Q*):** A K×N matrix where $q_{ki}$ is the proportion of individual *i*’s ancestry from ancestral population *k*, such that $0\leq q_{ki}\leq 1$ and $\sum_{k=1}^{K}q_{ki}=1$.
- **Allele frequencies (*F*):** An M×K matrix where $f_{jk}$ is the frequency of the alternate allele at SNP *j* in ancestral population *k*, such that $0\leq f_{jk}\leq 1$.

The model connects these by defining an expected individual-specific allele frequency $p_{ji}=\sum_{k=1}^{K}f_{jk}q_{ki}$, which represents the weighted average of the ancestral frequencies. The observed genotype $G_{ji}$ is then modeled as a random draw from a binomial distribution, $G_{ji}∼\text{Binomial}(2,p_{ji})$. The primary goal is to infer the latent matrices *Q* and *F* from the observed genotypes *G*.

### 2.2 Memory optimization

Because each observed genotype entry $G_{ji}$ takes only one of three discrete states, the dataset can be compactly encoded using low-precision formats. We utilize an 8-bit encoding for CPU computations and a 2-bit encoding for GPU computations, converting to a 64-bit format strictly for the data subset actively being processed. This approach reduces the data transfer overhead between the disk, CPU, and GPU while minimizing the overall memory footprint. A description of the memory requirements is detailed in Supplementary Note 1, available as supplementary data at *Bioinformatics* online.

### 2.3 Parameter estimation

The parameters of the model are estimated by maximizing the likelihood of the observed genotype data *G*. This is equivalent to minimizing the negative log-likelihood function. Assuming each genotype $G_{ji}$ is an independent draw from a binomial distribution, the negative log-likelihood is given by


(1) $$L(Q,F)=-\sum_{j=1}^{M}\sum_{i=1}^{N}\left[G_{ji}\;\text{log}(p_{ji})+(2-G_{ji})\;\text{log}(1-p_{ji})\right]$$

Subject to the parameter constraints, the optimization problem becomes


(2) $$\begin{array}{cccc} & \underset{Q,F}{\text{minimize}} & & L(Q,F)\\ & \text{subject}\;\text{to} & & 0\leq f_{jk}\leq 1,\;\forall j,k\\ & & & \sum_{k=1}^{K}q_{ki}=1,\;\forall i,\;q_{ki}\geq 0,\;\forall k,i \end{array}$$

### 2.4 Expectation–Maximization

We employ the EM procedure introduced in FRAPPÉ (Tang *et al.* 2005) and later adopted by ADMIXTURE, which iteratively updates the ancestry proportions *Q* and allele frequencies *F*.

In the Expectation step, we compute $a_{\mathrm{jik}}^{\left(t\right)}$ and $b_{\mathrm{jik}}^{\left(t\right)}$, the posterior probabilities that an observed alternate or reference allele, respectively, originated from ancestral population *k*:


(3) $$a_{\mathrm{jik}}^{\left(t\right)}=\frac{q_{ki}^{\left(t\right)}f_{jk}^{\left(t\right)}}{\sum_{m=1}^{K}q_{mi}^{\left(t\right)}f_{jm}^{\left(t\right)}}\;b_{\mathrm{jik}}^{\left(t\right)}=\frac{q_{ki}^{\left(t\right)}(1-f_{jk}^{\left(t\right)})}{\sum_{m=1}^{K}q_{mi}^{\left(t\right)}(1-f_{jm}^{\left(t\right)})}$$

In the Maximization step, the model parameters are updated by maximizing the expected complete-data log-likelihood with respect to the current posterior assignments:


(4) $$\begin{array}{c} f_{jk}^{\left(t+1\right)}=\frac{\sum_{i=1}^{N}G_{ji}a_{\mathrm{jik}}^{\left(t\right)}}{\sum_{i=1}^{N}\left[G_{ji}a_{\mathrm{jik}}^{\left(t\right)}+(2-G_{ji})b_{\mathrm{jik}}^{\left(t\right)}\right]}\\ q_{ki}^{\left(t+1\right)}=\frac{1}{2M}\sum_{j=1}^{M}\left[G_{ji}a_{\mathrm{jik}}^{\left(t\right)}+(2-G_{ji})b_{\mathrm{jik}}^{\left(t\right)}\right] \end{array}$$

This procedure alternates between computing expected ancestry assignments and updating parameter estimates until convergence.

### 2.5 Initialization

The choice of starting values for ancestry proportions *Q* and allele frequencies *F* is critical for ensuring convergence in EM-based admixture models. Naive random initialization often results in entrapment in suboptimal local maxima, a problem that is further exacerbated by accelerated algorithms.

While Neural Admixture (Dominguez Mantes *et al.* 2023) employs soft clustering on data projected via principal component analysis to provide informative initialization points, it struggles to scale to large numbers of ancestral populations or very large sample sizes. To address this, we adopt and refine the initialization strategy employed by SCOPE (Chiu et al. 2022) and fastmixture (Santander *et al.* 2024), incorporating both an advanced spectral decomposition and an alternating least squares (ALS) factorization framework. Specifically, we implement randomized singular value decomposition with dynamic shifts (Feng *et al.* 2024), a technique that utilizes dynamically shifted power iterations to refine the approximation of leading singular vectors.

The resulting low-rank structure approximates the centered genotype matrix, which serves as the target for the ALS factorization. This procedure jointly estimates the starting *Q* and *F* matrices prior to EM optimization. While SCOPE and fastmixture rely on naive box-clipping to project parameters in each iteration to satisfy the constraints in Equation (2), such an approach—which simply truncates out-of-bound parameters to the nearest edge of the [0,1] interval—fails to redistribute the remaining variables to account for the underlying dependency. This abrupt truncation displaces the solution from the true gradient path, inducing oscillatory convergence patterns or even preventing convergence altogether at a large number of populations or under conditions of high ancestral correlation.

To preserve the true gradient and strictly satisfy the Karush-Kuhn-Tucker (KKT) optimality conditions across the entire feasible region, we formulate the row-wise ALS updates as bounded variable least squares (BVLS) problems (Stark and Parker 1995). We solve these efficiently via a three-state variant of the block principal pivoting algorithm (Kim and Park 2011), which dynamically manages free variables alongside lower and upper active bounds to enforce the 0≤x≤1 constraints. After each update, to strictly satisfy the constraints formulated in Equation (2) and avoid numerical instabilities, *F* and *Q* are capped slightly inside the boundaries and the rows of *Q* are $L_{1}$-normalized, mapping admixture proportions onto the interior of the *K*-dimensional probability simplex. A detailed description of the initialization is provided in Supplementary Note 2, available as supplementary data at *Bioinformatics* online.

In order to demonstrate the empirical advantages of our initialization strategy, we evaluated its performance across a range of ancestral populations using 100 000 samples and 100 000 SNPs from the UK Biobank. Across all tested scenarios, our approach yields a significantly higher initial value of the objective function L(Q,F) compared to fastmixture, leading to faster convergence (Fig. 3) and improved final objective values. Furthermore, our initialization method consistently captures the expected scaling behavior with an increasing number of populations leading to higher log-likelihoods, as the larger parameter space accommodates finer ancestral resolution. Detailed comparisons of the initial and resulting log-likelihood values are provided in Supplementary Note 3, available as supplementary data at *Bioinformatics* online.

### 2.6 Adam-EM

Standard EM algorithms exhibit linear convergence rates, which become prohibitively slow on biobank-scale datasets. Established fixed-point acceleration schemes seek to bypass this bottleneck, but they often introduce substantial per-iteration overhead. For instance, the squared iterative method (SQUAREM) (Varadhan and Roland 2008) and the second-order quasi-Newton algorithms employed by ADMIXTURE and fastmixture (Zhou *et al.* 2009) typically necessitate two EM evaluations per parameter update to formulate their extrapolations or approximate the Hessian. Similarly, Anderson acceleration (Walker and Ni 2011) requires storing a history of previous residuals and solving a least squares problem at each iteration. These requirements effectively double the computational cost per step or introduce severe memory and processing bottlenecks in high-dimensional settings.

**Algorithm 1** Adam-EM.

**Require:**$\theta_{0}$, α, $\beta_{1},\beta_{2}$, ϵ

1: m←0, v←0, t←0

2: **while** not converged **do** 

3:  $\theta_{EM}\leftarrow \text{EM}(\theta_{t})$▹ Compute standard EM proposal

4:  $g_{t}\leftarrow \theta_{EM}-\theta_{t}$▹ Derive pseudo-gradient

5:  $m\leftarrow \beta_{1}m+(1-\beta_{1})g_{t}$

6:  $v\leftarrow \beta_{2}v+(1-\beta_{2})g_{t}^{2}$

7:  $\hat{m}\leftarrow m/(1-\beta_{1}^{t+1})$

8:  $\hat{v}\leftarrow v/(1-\beta_{2}^{t+1})$

9:  $\theta_{t+1}\leftarrow \theta_{t}+\alpha \frac{\hat{m}}{\sqrt{\hat{v}}+\epsilon }$▹ Apply adaptive update

10:  t←t+1

11: **end while** 

12: **return**$\theta_{t}$

To overcome this limitation, we introduce Adam-EM, an optimization method that integrates the stability of EM with the adaptive dynamics of the Adam optimizer. This approach uses first-order information, unlike second-order methods. The EM-induced parameter shift can be interpreted as a *pseudogradient*—a proxy for the steepest ascent direction on the likelihood manifold (Lange 1995). This interpretation is grounded in the fact that the EM update represents the true gradient transformed by a positive-definite matrix, effectively acting as a first-order method operating on a locally reshaped likelihood surface (Salakhutdinov *et al.* 2003). Feeding this *pseudogradient* into Adam allows adaptive moment estimation to normalize updates across parameters with different sensitivities. This effectively approximates curvature and injects momentum without the computational overhead of Hessian construction. The detailed pseudocode for this general optimization strategy is presented in Algorithm 1. In this generic formulation, the stopping criterion is application-specific, typically based on thresholding relative parameter changes or monitoring the target objective function.

Beyond genetic clustering and ancestry inference, our framework shows potential applicability to the broad class of latent variable models where EM updates are computationally expensive and exact gradients are intractable—such as probabilistic matrix factorization and topic modeling. Its underlying mechanism, which decouples the search direction (provided by EM) from the step size dynamics (managed by Adam), provides a theoretical foundation for its use in other settings characterized by high dimensionality and ill-conditioned parameter spaces. While further validation is needed beyond genetics, the method offers a scalable alternative that combines first-order stability with convergence speed that matches or exceeds second-order methods.

### 2.7 Optimization

We apply the Adam-EM acceleration scheme to simultaneously optimize the admixture proportions *Q* and allele frequencies *F*. In each iteration, we utilize the standard EM update rules derived in Equations (3) and (4) to compute the *pseudogradients*, following the logic described in Algorithm 1. After the adaptive step, parameters are projected to strictly satisfy the constraints formulated in Equation (2) by applying box-clipping to the [0,1] interval for both matrices, followed by $L_{1}$-normalization of the rows of *Q*. Moment buffers (m,v) are not reset upon parameter clipping, as potential boundary chattering is effectively mitigated by our step-size adaptation mechanism.

To promote numerical stability and efficient convergence, we incorporate a dynamic learning rate schedule that monitors the objective function L(Q,F) defined in Equation (1). Computing the log-likelihood is costly, and storing biobank-scale matrices for rollbacks incurs substantial memory overhead, so we save parameters only when the objective improves. Furthermore, to reduce floating-point error accumulation—particularly in momentum-based schemes (Higham 2002)—the objective is evaluated every *c* iterations. If no improvement occurs over ρ consecutive checks, the learning rate is reduced by a factor γ∈(0,1). Optimization halts safely once the learning rate reaches a minimum $\alpha_{\min}$. This strategy allows occasional likelihood-worsening steps within the *c*-iteration window but serves as a robust step-size heuristic, with subsequent iterations at the reduced rate refining the solution. The complete procedure is detailed in Algorithm 2, and all hyperparameters are provided in Supplementary Note 4, available as supplementary data at *Bioinformatics* online.

**Algorithm 2** ADAMIXTURE.

**Require:** $G\in R^{M\times N},K,\alpha ,\gamma ,\beta_{1},\beta_{2},\epsilon ,Q,F,t_{\text{max}},\tau ,\rho ,c,\alpha_{\min}$

1: $m_{Q}=v_{Q}=m_{F}=v_{F}\leftarrow 0$

2: t←0

3: $L_{\text{best}}\leftarrow -\infty ,\;c_{\text{stall}}\leftarrow 0$

4: $Q_{\text{best}}\leftarrow Q,\;F_{\text{best}}\leftarrow F$

5: **while**$t<t_{\text{max}}$**and**$\alpha >\alpha_{\min}$**do** 

6:  $Q_{1},F_{1}\leftarrow \text{EM}(G,Q,F)$

7:  $\nabla_{Q}\leftarrow Q_{1}-Q,\;\nabla_{F}\leftarrow F_{1}-F$

8:  **for**θ∈{Q,F}**do** 

9:     $m_{\theta }\leftarrow \beta_{1}m_{\theta }+(1-\beta_{1})\nabla_{\theta }$

10:   $v_{\theta }\leftarrow \beta_{2}v_{\theta }+(1-\beta_{2}){\left(\nabla_{\theta }\right)}^{2}$

11:   ${\hat{m}}_{\theta }\leftarrow m_{\theta }/(1-\beta_{1}^{t+1})$

12:   ${\hat{v}}_{\theta }\leftarrow v_{\theta }/(1-\beta_{2}^{t+1})$

13:   $\theta \leftarrow \theta +\alpha \frac{{\hat{m}}_{\theta }}{\sqrt{{\hat{v}}_{\theta }}+\epsilon }$

14:  **end for** 

15:  **if**(t+1)mod c=0**then** 

16:   $L_{\text{curr}}\leftarrow L(Q,F)$

17:   **if**$L_{\text{curr}}>L_{\text{best}}+\tau$**then** 

18:    $L_{\text{best}}\leftarrow L_{\text{curr}}$

19:    $Q_{\text{best}},F_{\text{best}}\leftarrow Q,F$

20:    $c_{\text{stall}}\leftarrow 0$

21:   **else** 

22:    $c_{\text{stall}}\leftarrow c_{\text{stall}}+1$

23:    **if**$c_{\text{stall}}\geq \rho$**then** 

24:     $\alpha \leftarrow \max(\alpha \cdot \gamma ,\alpha_{\min})$

25:     $c_{\text{stall}}\leftarrow 0$

26:    **end if** 

27:   **end if** 

28:  **end if** 

29:  t←t+1

30: **end while** 

31: **return**$Q_{\text{best}},F_{\text{best}}$

### 2.8 Benchmarking

#### 2.8.1 Assessment of results

We assess method performance through a strategy encompassing quantitative goodness-of-fit metrics, reproducibility analysis, and qualitative inspection of population structure. Given the unsupervised paradigm of genetic ancestry inference, where absolute ground truth is absent in empirical datasets, we rely on the negative log-likelihood from Equation (1) as the primary objective function. This metric quantifies the statistical fidelity of the parameter estimates and the model’s capacity to explain the observed genetic variation.

To evaluate the robustness of the inferred ancestry components across independent initializations, we measure cluster stability by computing the mean pairwise Pearson correlation and the Frobenius distance between *Q* matrices over five independent runs per configuration. This provides a reproducibility check, ensuring that the results are consistent and not artifacts of local minima.

Complementing statistical fit and stability, we benchmark computational efficiency by recording wall-clock runtime across varying dataset dimensions to evaluate scalability and qualitatively validate the results by visualizing the *Q* matrices using pong (Behr *et al.* 2016). All results from empirical and simulated datasets are publicly available on figshare.

#### 2.8.2 Simulated datasets

To enable direct comparison with state-of-the-art acceleration methods, we use the simulation benchmarks introduced by fastmixture (Santander *et al.* 2024), generated via coalescent simulations with a constant effective population size of 10 000 to model neutral evolution.

We consider four demographic scenarios (A-D). Scenarios A, B, and C represent discrete populations with constant sizes, while Scenario D models a complex American-Admixture event (Browning *et al.* 2018). Sample sizes are 1000 for Scenarios A, B, and D, and 1600 for Scenario C. After filtering for minor allele frequency (MAF) >0.05, the resulting datasets contain between 500 000 and 700 000 SNPs.

#### 2.8.3 Empirical datasets

To evaluate ancestry inference under realistic conditions, we selected four genomic cohorts representing distinct regimes of sample size, SNP density, and population structure. This diverse set provides a robust framework for assessing both scalability and accuracy, capturing patterns from fine-scale stratification to global population divergence.

The **UK Biobank (UKB)** (Bycroft *et al.* 2018) serves as our primary benchmark for biobank-scale inference. For the central accuracy and runtime comparisons, we curated a standard subset comprising 100 000 individuals and 100 000 SNPs, obtained via random subsampling and MAF filtering. To systematically evaluate algorithmic scalability across different data dimensions, we also generated auxiliary subsets with sample sizes ranging from 50 000 to 500 000 individuals and SNP counts from 100 000 to 500 000. Predominantly of European ancestry, this cohort challenges both scale and fine-structure resolution, reflecting modern genome-wide association study (GWAS) contexts.

The **Domestic Dog** dataset (Parker *et al.* 2017) includes 1355 samples from 166 breeds genotyped at 150 131 SNPs. Originating from lineages across all inhabited continents, the cohort exhibits strong genetic drift and sharp population boundaries due to breed isolation. It provides a rigorous test of model robustness and adaptability, particularly for inferring high numbers of ancestral populations.

The merged dataset of the **Human Genome Diversity Project (HGDP)** and **1000 Genomes Project** comprises 4091 individuals from globally distributed populations. After MAF filtering, the panel includes ∼500 000 autosomal SNPs, with sex chromosomes excluded. Compared to UK Biobank, this panel captures substantial worldwide genetic diversity and pronounced population structure, making it a complementary benchmark for evaluating performance under complex, multiancestry scenarios (Li *et al.* 2008, Consortium *et al.* 2015).

#### 2.8.4 Experimental setup

All experiments were conducted on the UCSC Genomics Institute compute cluster. Each computational run was allocated 64 logical cores on an AMD EPYC 7713 processor. As Neural Admixture and the GPU implementation of ADAMIXTURE are the only methods capable of leveraging hardware acceleration, they were each provisioned with a single NVIDIA A100 GPU (80 GB VRAM); all other methods were restricted to CPU execution.

We benchmark ADAMIXTURE against ADMIXTURE, Neural Admixture, fastmixture, and SCOPE, with details on software versions, libraries, and execution parameters provided in Supplementary Note 5, available as supplementary data at *Bioinformatics* online.

## 3 Results

### 3.1 Performance on simulated benchmarks

Quantitative benchmarking on simulated data confirms that ADAMIXTURE consistently recovers the global maximum with numerical precision and stability comparable to or exceeding that of ADMIXTURE and fastmixture. While all three likelihood-based methods converge to a very similar global mode, our method achieves marginally higher absolute log-likelihood values across all simulated datasets, demonstrating comparable convergence tightness to the baselines (Table 1).

**Table 1:** Log-likelihood measures on simulated datasets.^a^

Method	A	B	C	D
ADAMIXTURE	**−637 729 492.3 (0.1)**	**−629 360 597.6 (0.0)**	**−1 006 294 343.7 (0.1)**	**−429 286 152.3 (0.1)**
fastmixture	**−**637 729 494.0 (0.7)	**−**629 360 599.1 (0.3)	**−**1 006 294 347.8 (0.5)	**−**429 286 152.5 (0.1)
Neural Admixture	**−**637 765 240.0 (3.6 × 10^4^)	**−**630 345 588.3 (8.4 × 10^5^)	**−**1 007 821 071.2 (6.6 × 10^3^)	**−**429 332 568.9 (2.9 × 10^4^)
ADMIXTURE	**−**637 729 492.4 (0.5)	**−**629 360 598.5 (1.6)	**−**1 006 294 347.6 (4.1)	**−**429 286 155.6 (4.1)
SCOPE	**−**637 846 223.5 (10.2)	**−**629 538 208.7 (38.1)	**−**1 006 604 603.8 (244.3)	**−**429 584 894.3 (84.4)

a Each dataset was run with the specified number of clusters: Dataset A (K=3), B (K=4), C (K=5), D (K=3). For each method, the mean of five independent runs is reported, with the standard deviation shown in parentheses. Bold values indicate the best log-likelihood achieved for each dataset.

In contrast, approximate methods fail to reach this optimality. SCOPE consistently produces suboptimal fits, falling short of the likelihood quality achieved by EM-based approaches. Similarly, Neural Admixture exhibits high variance across replicates, yielding unreliable parameter estimates.

### 3.2 Performance on empirical benchmarks

Evaluation on large-scale empirical cohorts further validates the efficacy of our framework. As detailed in Table 2, ADAMIXTURE attains the maximum log-likelihood across all evaluated datasets. Crucially, our method demonstrates superior numerical stability, maintaining low standard deviations across replicates, whereas competing accelerated methods frequently exhibit substantial variance. Across all cohorts, Neural Admixture and SCOPE systematically fail to reach the optimal mode, yielding model fits with likelihoods orders of magnitude worse than those of state-of-the-art methods.

**Table 2:** Log-likelihood measures on empirical datasets.^a^

Method	UKB	Dogs	HGDP
ADAMIXTURE	**−5 324 953 196.1 (21.4)**	**−194 648 323.0 (1.6 ** × **10^4^)**	**−1 131 620 423.1 (0.2)**
fastmixture	**−**5 325 159 277.5 (3.2 × 10^5^)	**−**194 651 453.1 (1.1 × 10^4^)	**−**1 131 620 449.9 (6.5)
Neural Admixture	**−**5 334 181 181.0 (1.3 × 10^5^)	**−**195 266 696.7 (8.4 × 10^4^)	**−**1 132 157 396.4 (3.5 × 10^5^)
ADMIXTURE	**−**5 324 953 302.9 (32.0)	**−**194 749 866.8 (6.4 × 10^4^)	**−**1 131 908 125.6 (5.0 × 10^5^)
SCOPE	**−**5 340 156 808.4 (3.5 × 10^6^)	**−**195 985 480.7 (63.4)	**−**1 134 190 154.7 (2.8 × 10^3^)

a Each dataset was run with the specified number of clusters: UKB (K=5), DOGS (K=20), HGDP (K=8). For each method, the mean of five independent runs is reported, with the standard deviation shown in parentheses. Bold values indicate the best log-likelihood achieved for each dataset.

**Figure 1:**
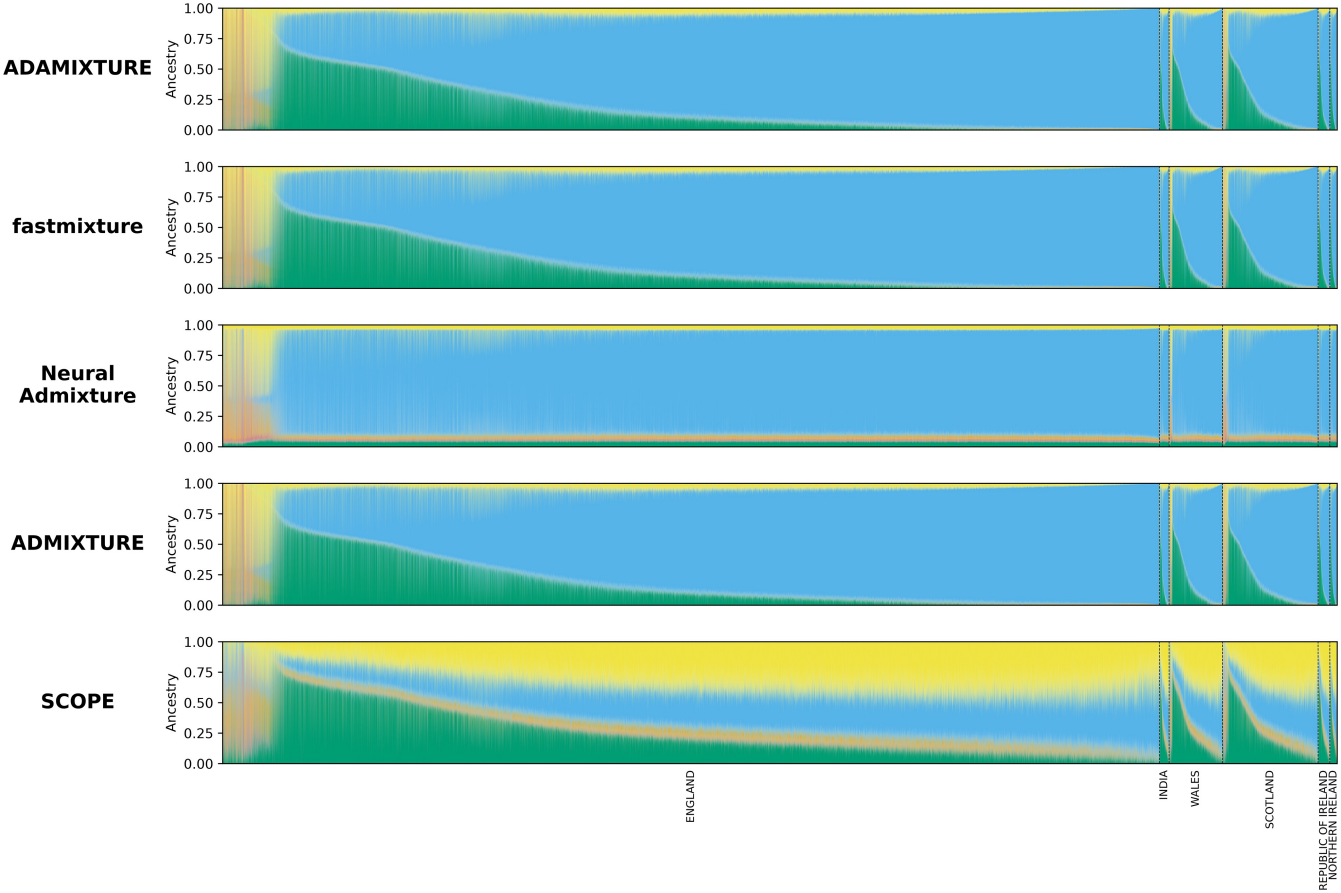
Visualization of the estimated ancestry proportions for the UK Biobank dataset (K=5) obtained using the different benchmarked methods. Each individual is represented by a thin vertical line partitioned into colored segments that denote their estimated fractional membership in each of the *K* ancestral clusters. For each method, the displayed results correspond to the run achieving the highest log-likelihood among five independent runs. Labels correspond to self-reported location of birth. Only regions with a sufficient number of individuals to ensure clear visualization are shown; these include England, India, Wales, Scotland, the Republic of Ireland, and Northern Ireland.

While fastmixture and classical ADMIXTURE generally converge toward near-optimal solutions, our method consistently outperforms both baselines in precision and convergence robustness. The practical impact of this improved robustness is illustrated in Fig. 1. Qualitative inspection shows that the ancestry proportions inferred by ADAMIXTURE are visually indistinguishable from those recovered by fastmixture and classical ADMIXTURE, demonstrating that the proposed acceleration strategy preserves fine-scale population structure.

Conversely, likelihood-free accelerators introduce significant artifacts. Under default settings, Neural Admixture oversmooths estimates, assigning similar ancestry proportions to distinct subpopulations—a behavior reminiscent of mode collapse or aggressive regularization. Although neural methods are highly sensitive to parameter tuning and may improve with extensive optimization, these default-setting inaccuracies corroborate prior reports of suboptimal neural-network fits (Santander *et al.* 2024). On the other hand, SCOPE yields noisy clusters that obscure underlying genetic signals. Notably, SCOPE’s solutions closely resemble those obtained using only our spectral initialization, without EM refinement. This indicates that its ALS-based strategy may stagnate at a superficial local minimum, explaining the fast runtime but substantially lower solution quality.

### 3.3 Quantitative stability and precision

To evaluate the reliability of inferred population structures, we analyzed cluster stability and reconstruction error across independent runs. As detailed in Supplementary Note 6, available as supplementary data at *Bioinformatics* online, ADAMIXTURE demonstrates exceptional robustness, maintaining a mean pairwise Pearson correlation of ≈1.0 across replicates for all datasets.

In terms of numerical precision, our method achieves exceptionally low Frobenius distances, outperforming likelihood-free accelerators in most scenarios while matching or exceeding the precision of likelihood-based implementations. These findings confirm that the proposed acceleration strategy does not compromise model fidelity, demonstrating robustness to the stochasticity introduced during the initialization phase.

### 3.4 Runtime analysis

**Figure 2:**
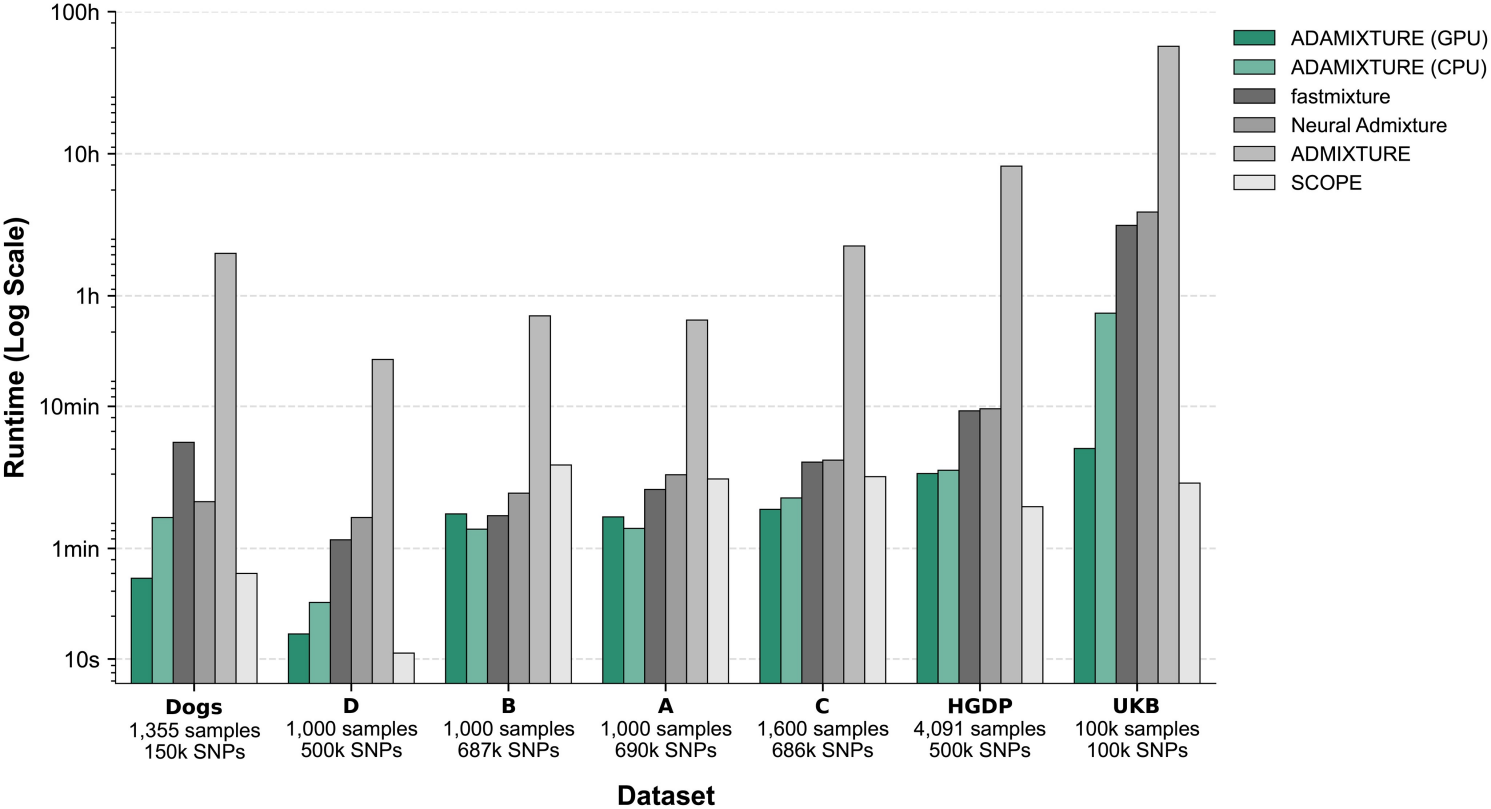
Comparison of runtime performance across synthetic (A-D) and empirical (Dogs, HGDP, UKB) datasets. The *y*-axis is shown on a logarithmic scale to accommodate the extreme variance in execution times. Results represent the mean of five independent runs.

The runtime efficiency of ADAMIXTURE was systematically benchmarked against competing methods across all evaluated datasets, achieving substantial acceleration over classical methods (Fig. 2). The most substantial performance gain is observed when compared to the standard ADMIXTURE algorithm on the largest empirical dataset (100 000 samples). While ADMIXTURE requires over 57 h to reach convergence, our baseline CPU implementation completes the inference in approximately 45 min. Crucially, the introduction of hardware acceleration via ADAMIXTURE (GPU) further reduces this runtime to a mere 5 min, achieving a staggering 680× overall speedup. This effectively transforms the analysis of biobank-scale cohorts from a multiday task into a subhour routine.

On smaller cohorts, the CPU-GPU performance gap is narrow because initialization dominates execution time in low-complexity regimes. However, as dataset dimensions scale up, the GPU-accelerated Adam-EM optimization becomes the defining factor, as demonstrated in the subsequent sections.

Furthermore, ADAMIXTURE consistently outperforms other modern accelerated frameworks. On average across all evaluated cohorts, it achieves a 3.5× speedup over fastmixture and a 4× speedup over Neural Admixture. A nuanced profile emerges for SCOPE, which exhibits the lowest absolute wall-clock times on datasets with moderate feature dimensionality (<500 000 SNPs). Nevertheless, both ADAMIXTURE variants prove more computationally efficient on high-dimensional datasets (synthetic sets A-C). Importantly, as established in our prior analyses, SCOPE’s speed advantage in some scenarios is consistently achieved at the expense of solution quality.

### 3.5 Scalability analysis

To ensure a rigorous assessment of computational efficiency, we restrict our scalability benchmarks to ADAMIXTURE and fastmixture. Heuristic approximations such as SCOPE and Neural Admixture are excluded from this direct comparison because, as demonstrated in Tables 1 and 2, they consistently fail to converge to the global maximum; their lower runtimes derive in part from premature termination at suboptimal optima rather than fully from algorithmic efficiency. Moreover, ADMIXTURE is omitted due to its prohibitive computational cost. As shown in Fig. 2, its execution on a UK Biobank subset (K=5) already approaches 60 h, rendering it intractable for the configurations explored in this evaluation.

We evaluate how the number of ancestral populations affects processing time using a fixed subset of the UK Biobank (100 000 samples, 100 000 SNPs). For this benchmark, the maximum iteration limit for both methods was increased to 10 000, as the default limits were insufficient to guarantee convergence for high values of *K*. Additionally, some hyperparameters for ADAMIXTURE were adjusted, with details provided in Supplementary Note 4, available as supplementary data at *Bioinformatics* online.

**Figure 3:**
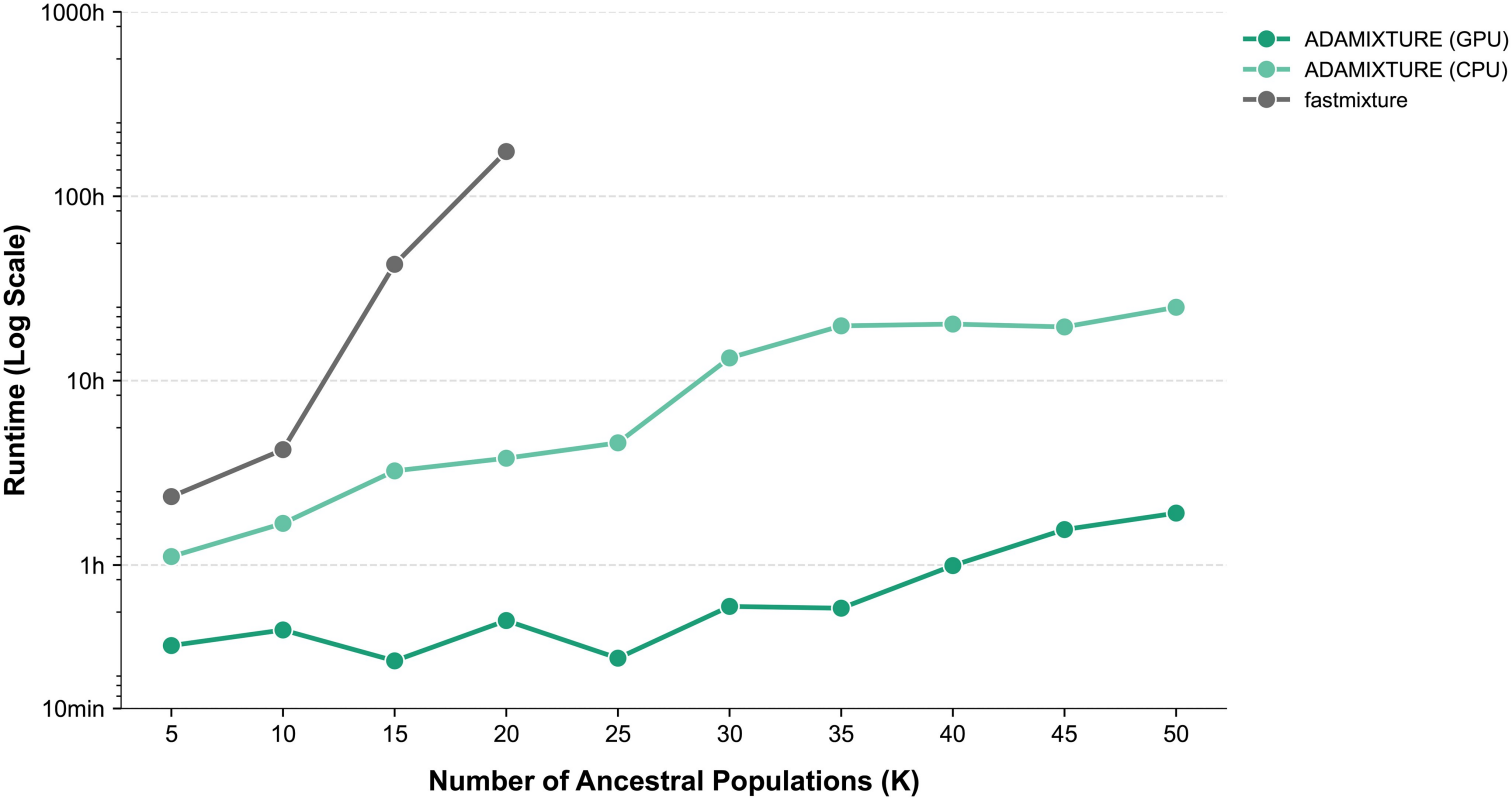
Runtime scalability with respect to the number of ancestral populations (*K*) on a subset of the UK Biobank dataset (100 000 samples, 100 000 SNPs). Results represent the mean of five independent runs. Data points for fastmixture at K≥25 are omitted as they exceeded a 10-day runtime limit.

As illustrated in Fig. 3, ADAMIXTURE (CPU) exhibits profoundly superior performance scaling compared to fastmixture. The execution of fastmixture becomes impractical as the number of ancestral populations increases, with runs for K≥25 failing to complete within a 10-day limit. In contrast, our CPU version demonstrates near-logarithmic execution growth across the entire evaluated range up to K=50.

This efficiency gain is intrinsic to our Adam-EM formulation. By eliminating the necessity for the traditional two-step EM procedure, our method effectively halves the processing overhead associated with updating the population matrices at each step. Importantly, this drastic reduction in per-iteration cost is not offset by a need for more iterations. As shown in Supplementary Note 3, available as supplementary data at *Bioinformatics* online, ADAMIXTURE converges in a comparable or lower number of iterations while simultaneously reaching better solutions.

Additionally, the GPU implementation of ADAMIXTURE further reduces execution time by an order of magnitude. Even at high complexity (K=50), convergence occurs in approximately 2 h, making previously prohibitive biobank-scale clustering tasks routine.

**Figure 4:**
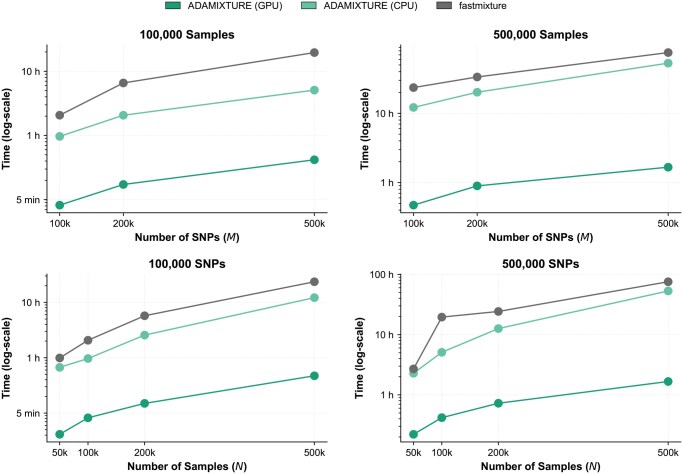
Scalability with respect to dataset dimensions. The plot illustrates runtime growth on the UK Biobank dataset as a function of the number of SNPs (top panels) and sample size (bottom panels), with the number of populations fixed at K=5. Results represent the mean of five independent runs.

Beyond the number of ancestral populations, we also assess data scaling by varying the number of samples and SNPs while maintaining a fixed number of populations. As illustrated in Fig. 4, ADAMIXTURE (CPU) consistently outperforms fastmixture across all evaluated configurations. Moreover, ADAMIXTURE (GPU) achieves a paradigm shift in execution speed, dramatically accelerating convergence. At the maximum evaluated scale, the GPU version converges in under 2 hours, whereas the baseline fastmixture requires nearly 100 hours. This performance gap highlights ADAMIXTURE’s exceptional suitability for massive cohorts, offering a fast, robust solution that scales naturally without compromising statistical rigor.

Taken together, these results demonstrate that the primary processing bottleneck for standard CPU-based EM methods lies in the number of estimated populations rather than the raw dimensions of the genotype data. While scaling samples and markers yields a predictable, manageable increase in execution time, increasing the number of latent populations severely slows traditional algorithms. It is precisely in overcoming this fundamental limitation that the GPU-accelerated version of ADAMIXTURE demonstrates its greatest advantage, seamlessly absorbing the workload of high-dimensional clustering.

## 4 Discussion

This study demonstrates that integrating first-order adaptive optimization with the EM framework effectively resolves the scalability bottlenecks currently plaguing genetic clustering. Our findings indicate that the heavy computational expense of second-order updates is redundant in high-dimensional genomic settings, where adaptive momentum can achieve comparable convergence tightness with significantly lower overhead.

Our results underscore that the precision of the initialization phase is as critical as the optimization itself. While previous accelerators relied on heuristic clipping that often derailed the initial gradient path, the shift toward an exact BVLS formulation ensures that the optimization consistently begins within the basin of the global maximum.

Recognizing that the analysis of full-scale biobanks still poses a nontrivial time investment on standard CPU hardware, our framework is natively designed to harness massive hardware parallelism. Through its GPU-accelerated implementation, we effectively bridge the gap between statistical guarantees and ultrafast execution. While prior attempts like Neural Admixture utilized GPUs but sacrificed likelihood maximization for speed, our approach achieves the optimal model fit in a fraction of the time. This delivers an additional order-of-magnitude reduction in runtime, compressing biobank-scale analyses that previously required days or weeks into the order of an hour, even at extreme levels of complexity.

Looking forward, on the computational front, extending our implementation to multi-GPU architectures and mixed-precision computing presents a straightforward pathway to further accelerate inference for unprecedented cohort sizes, although special care must be taken to mitigate numerical imprecision introduced by distributed gradient reductions and reduced-precision formats. In terms of downstream applications, a critical next step will be evaluating the impact of these highly resolved *Q*-matrices on population stratification correction in complex trait GWAS, as well as improving the cross-population portability of polygenic risk scores. Furthermore, adapting the Adam-EM framework to operate on haplotype clusters, which have recently demonstrated superior accuracy in ancestry estimation compared to unlinked SNPs (Meisner 2026), or extending it to accelerate local ancestry inference, represents a highly relevant methodological extension. Finally, exploring the integration of this optimizer with models designed for low-coverage sequencing data or ancient DNA could also broaden its utility.

In summary, ADAMIXTURE represents a pivotal advancement in the analysis of large-scale genomic data. By coupling the statistical foundation of maximum-likelihood estimation with modern adaptive optimization, BVLS initialization, and hardware acceleration, we have eliminated the historical necessity to compromise between computational feasibility and scientific precision. As biobank cohorts expand to millions of individuals, the ability to perform robust and scalable inference becomes a prerequisite for discovery. Our method fills this critical gap, providing a tool for uncovering fine-scale population structures that underpin our understanding of human genetic variation, demographic history, and complex disease architecture.

## Supplementary Material

btag236_Supplementary_Data

## Data Availability

Data from the 1000 Genomes Project (1kGP) and Human Genome Diversity Project (HGDP) used in this study are available from https://gnomad.broadinstitute.org/downloads#v3-hgdp-1kg as published in https://doi.org/10.1101/gr.278378.123 and the dogs data used in this study were published in https://doi.org/10.1016/j.celrep.2017.03.079 and are available via GEO: GSE90441, GSE83160, GSE70454, and GSE96736. The UK Biobank data used in this study are available to all bona fide researchers through the UK Biobank Access Management System pending application and Research Ethics Committee approval.
